# Genetic therapies for cardiomyopathy: survey of attitudes of the patient community for the CureHeart project

**DOI:** 10.1038/s41431-024-01660-5

**Published:** 2024-07-07

**Authors:** Elizabeth Ormondroyd, Christopher Grace, Wendy Borsari, Anuj Goel, Barbara McDonough, Joel Rose, Christine Seidman, Hugh Watkins

**Affiliations:** 1 Division of Cardiovascular Medicine, Radcliffe Department of Medicine, University of Oxford, Oxford, UK; 2 NIHR Biomedical Research Centre, Oxford, UK; 3 CureHeart Patient Advisor, Plymouth, MA USA; 4 Harvard Medical School Department of Genetics, Boston, MA USA; 5 Cardiovascular Division, Brigham and Women’s Hospital, Boston, MA USA; 6 Cardiomyopathy UK, Amersham, UK; 7 Howard Hughes Medical Institute, Chevy Chase, MD USA

**Keywords:** Genetics research, Social sciences

## Abstract

Cardiomyopathies are a group of inherited heart muscle disorders. Expressivity is variable and while sometimes mild, complications can result in sudden cardiac death (SCD) at any age, heart failure and stroke. In around a third of patients a monogenic cause is identifiable, and development of genetic therapies that aim to correct the underlying genetic defect is underway. Here we describe results of a survey designed to understand preliminary views of the patient community about genetic therapies in the context of disease burden. The internet survey was publicized with a bespoke information video via patient support groups in the UK and USA; 634 people responded of whom 96% had a personal and/or family history of cardiomyopathy. Findings show that concern about cardiomyopathy-related issues with a future dimension, such as disease progression, is significantly greater than concern about current issues. A total of 93.6% thought that genetic therapies should be developed for cardiomyopathy. A majority would consider participation in a genetic therapy trial in six scenarios varying by age and clinical situation significantly more in the scenario of an adult with symptomatic disease and evident progression than an asymptomatic adult with SCD risk, or a child. In all scenarios, a majority said that the chance genetic therapy would stop or slow progression, and risk of serious adverse and unintended effects, were important considerations. Qualitative analysis of free-text responses found that concern was often informed by family experience. Patient consideration of genetic therapy is likely to require individualized assessment of the benefits and risks.

## Introduction

Cardiomyopathies are a clinically and genetically heterogeneous group of conditions affecting the structure and function of the heart [1]. They include hypertrophic cardiomyopathy (HCM), dilated cardiomyopathy (DCM), arrhythmogenic (right ventricular) cardiomyopathy ARVC/ACM and restrictive cardiomyopathy (RCM). Up to one in 250 people are affected [2, 3]. Symptoms result from impaired cardiac function or cardiac arrhythmia and can include dyspnea (shortage of breath), chest pain, dizziness, fatigue, syncope (cardiac faints), and cardiac arrest, although many patients have no or only mild symptoms. Complications including cardiac death, stroke, and heart failure, can occur at any age including in children and young people. Cardiomyopathies account for half of all cardiac transplants [4–6]. Symptoms can onset across the lifespan, however risk of dangerous arrhythmias and cardiac arrest are possible in the absence of symptoms. Risk management interventions and lifestyle restrictions such as avoidance of intense activity may be recommended for patients at any age and regardless of symptoms, and they may be excluded from competitive sport or regulated occupations.

In families affected by cardiomyopathy, individuals may progress at any age from becoming aware of their potential risk, being well but at known genetic risk of disease development, being clinically affected but asymptomatic, to requiring medical intervention to alleviate symptoms or life-threatening arrythmia. Individuals with an inherited cardiomyopathy may have impaired quality of life; cardiomyopathy can significantly impact patients’ employment, life planning, physical functioning and mental health [7, 8]. Patients may experience anxiety, a sense of isolation, and guilt about transmission [9]; younger age and implantable cardioverter defibrillator (ICD) shock increase risk for psychosocial difficulties [10]. A sudden death—often in a young apparently healthy person—may be the first indication of an inherited cardiomyopathy. Sudden cardiac death (SCD) has a major impact on families; grief is often profound and long term, with around half of first-degree relatives suffering clinically significant post-traumatic stress symptoms or prolonged grief [11, 12]. The need of relatives to understand the cause of death, and prevent a further SCD, motivates initiation of post mortem genetic testing [13], but psychosocial consequences of grief may complicate adaptation to findings [14].

An underlying monogenic cause is identifiable in around a third cardiomyopathy patients referred for genetic testing [15, 16]. Heterozygous cardiomyopathy mutations disturb normal biomechanical properties, promote aberrant intra- and inter-cell signaling and accelerate cardiomyocyte death, resulting in insidiously progressive abnormalities of myocardial architecture and function [5]. Common genetic variation and comorbidities have recently been shown to contribute to susceptibility and expression of HCM and DCM [17, 18]. Most cardiomyopathy-associated genes are located on autosomes and follow a dominant pattern of inheritance implying 50% risk to first degree relatives. Clinical genetic testing, usually of a phenotype-directed gene panel, is recommended following a diagnosis of cardiomyopathy [19]. An identified pathogenic variant can be cascaded to at-risk relatives, although expressivity is variable even within a family sharing the same genetic variant. Worry about children’s risk can be significant [20], and the decision to undergo pre-symptomatic testing is often motivated by a wish to know whether relatives, particularly descendants, are at risk [20–22]. Relatives’ perceptions of disease severity are influenced by personal and family experience, but perceptions of personal risk can be low in the absence of symptoms and active lifestyle, and some test to “rule out” risk [20, 22]. Psychological distress after pre-symptomatic testing is significantly associated with mismatch between subjective risk perception and result [22].

Lifelong care for patients and at-risk relatives includes periodic reassessment and SCD risk stratification [23] using cardiac imaging and ECG; management includes medical control of symptoms, ICD to abort life-threatening arrhythmias, lifestyle modification, septal reduction in HCM, catheter ablation and as a last resort, cardiac transplantation [24]. All currently available therapies fail to prevent disease progression, are incompletely effective and costly, and carry significant clinical risk and psychosocial burden.

Genetic therapies are becoming a realistic prospect for monogenic disorders [25] including cardiomyopathy, with progress recently made to correct pathogenic cardiomyopathy variants in animal models and human-derived cardiomyocytes [26, 27]. Challenges include gene delivery to the heart, and the heterogeneity of genes involved as well as disease mechanisms: pathogenic gene variants mediate disease either through dominant negative effects or haploinsufficiency. For dominant negative alleles, approaches might include allele-specific knockdown or antisense oligonucleotide silencing, CRISPR-Cas9, base or prime editing [28, 29]. For variants causing cardiomyopathy by haploinsufficiency, exogenous gene supplementation or upregulation of endogenous gene expression would be required.

Since genetic therapies bring a range of practical and ethical ramifications [30], patient, family and public input, including acceptability, will be vital for successful adoption. As part of a funding application (later funded as CureHeart, a multinational research partnership that aims to develop transformative and potentially curative gene therapy approaches for inherited cardiomyopathies [31]), we sought to understand patient and family perspectives of living with cardiomyopathy, and attitudes towards specific aspects of genetic therapies for cardiomyopathy.

## Methods

### Design and distribution

We developed an online survey using JISC (https://jisc.onlinesurveys.ac.uk), informed by existing literature, the authors’ research, clinical and personal experiences and those of patient representatives of Cardiomyopathy UK. Two patient involvement (PPI) meetings were carried out by remote meeting platform in August 2020, attended by a total of 10 affected Cardiomyopathy UK members. Meetings were moderated by a PPI professional and attended by EO and HW. Both sessions began with an introduction to genetic therapy and proceeded to elicit panel members’ thoughts about issues they felt important about living cardiomyopathy. The survey was piloted with a small number of patients in Oxford and Boston-based clinical inherited cardiac conditions services, and WB. The final survey (Supplementary Material) included closed, Likert, and vignette questions, and collected demographic and genetic testing information, elicited level of concern about factors that can be involved in living with cardiomyopathy, and respondent views about the importance of several genetic therapy attributes in six scenarios. Free text responses were also collected. In parallel, we developed an information video with PPI input, providing an overview of cardiomyopathy genetics and inheritance, the goals of genetic therapy, and development of genetic correction techniques. A link to the video was included at the start of the survey. The survey was publicized on the Cardiomyopathy UK website and ran from June–October 2021. It was then publicized via the Hypertrophic Cardiomyopathy Association (HCMA; https://4hcm.org/) and re-opened for a further 2 months. In the UK, the survey was considered not subject to the Department of Health’s UK Policy Framework for Health and Social Care Research (2017). In the US, IRB approval was obtained.

### Data analysis

Quantitative data were analyzed using SPSS and R, version 4.3.2. For Question 9 (How concerned are you about the following factors that can be involved in living with cardiomyopathy?), 20 issues were presented in the survey with possible responses on a four-point Likert scale from “very” to “not” concerned. Issues were assigned to one of three categories: “present” (seven issues), “future” (five issues) and “both present and future” (eight issues), according to timing of issue arising (Table S1). To determine whether there were differences between present and future issues, each issue listed in the survey was coded 1 to 4 in order of level of concern (not concerned to very concerned) and the following analyses were performed: the means, SDs, maximum and minimum values for “present”, “future” and “both” issues were calculated, and the distributions of the means plotted. Finally differences in the distribution of the means for present and future questions was tested using a Wilcoxon rank sum exact test. Free-text responses were analyzed using NVIVO 12. Thematic analysis [32] of responses to the two free text questions was undertaken using qualitative techniques including systematic iterative coding and development of themes within the responses. Selected free-text supporting the data analysis are shown in italics, and further free-text data in Table S2.

## Results

### Respondents

A total of 634 people responded, 61% female. In total, 4.8% said they identify as an ethnic minority, and 2.1% were unsure. Respondents lived in the UK (81%), USA (11.7%), or another country (7.3%), including Canada (*n* = 9), Australia (*n* = 5), Germany (*n* = 3), Sweden (*n* = 3), Spain (*n* = 2), India (*n* = 2), Israel (*n* = 2), Portugal (*n* = 2), Cyprus (*n* = 1), Greece (*n* = 1); Czech republic (*n* = 1); Philippines (*n* = 1), Mauritius (*n* = 1), South Sudan (*n* = 1), Pakistan (*n* = 1), Russian federation (*n* = 1), Palestine (*n* = 1), Netherlands (*n* = 1), Ukraine (*n* = 1), Austria (*n* = 1).

**Fig. 1:**
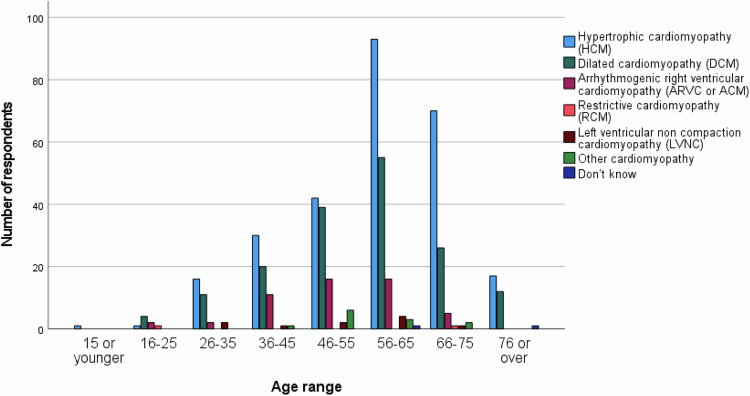
Cardiomyopathy phenotype of respondents. Graph shows the phenotype of respondents with cardiomyopathy, by age range.

Eighty-one percent of respondents said they have cardiomyopathy, 38% that someone in their family has cardiomyopathy, 3.5% that someone they know well outside the family has cardiomyopathy (more than one response was possible), and 0.5% said they were neither affected nor knew someone with cardiomyopathy. Median age range of all respondents was 56–65 years; 151 (23.8%) were aged 45 or under. Among people personally affected with cardiomyopathy (*n* = 514), the most common type in all age ranges was HCM, except age 16–25; the majority of respondents aged 16–25 with cardiomyopathy had DCM (Fig. 1).

Among respondents who have cardiomyopathy, 70.6% said that genetic testing had been done, while 24.3% said genetic testing had not been offered; 3.5% did not know and 1.4% said they had declined genetic testing.

### Concern about aspects of cardiomyopathy

**Fig. 2:**
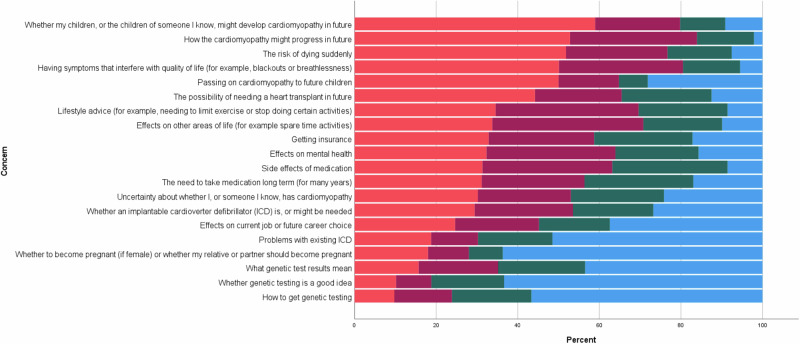
Concern about issues relating to cardiomyopathy. Proportion of respondents who selected ‘not concerned’ (blue); slightly concerned (green); quite concerned (purple); very concerned (orange), in reponse to the concerns shown.

The survey presented 20 issues that can be involved in living with cardiomyopathy. Issues presented and Likert scale responses per issue are shown in Fig. 2. Around half of respondents answered that they were “very concerned” about “whether my children, or the children of someone I know, might develop cardiomyopathy in future” (58.8%), “how the cardiomyopathy might progress in the future,” (52.5%), “the risk of dying suddenly” (51.4%), “having symptoms that interfere with quality of life” (50.2%), passing on cardiomyopathy to future children (49.3%), and the possibility of needing a heart transplant in the future (44%). For Question 9, no single concern was answered by all respondents; the number of respondents per concern ranged from 567 to 624 (Table S1).

**Fig. 3:**
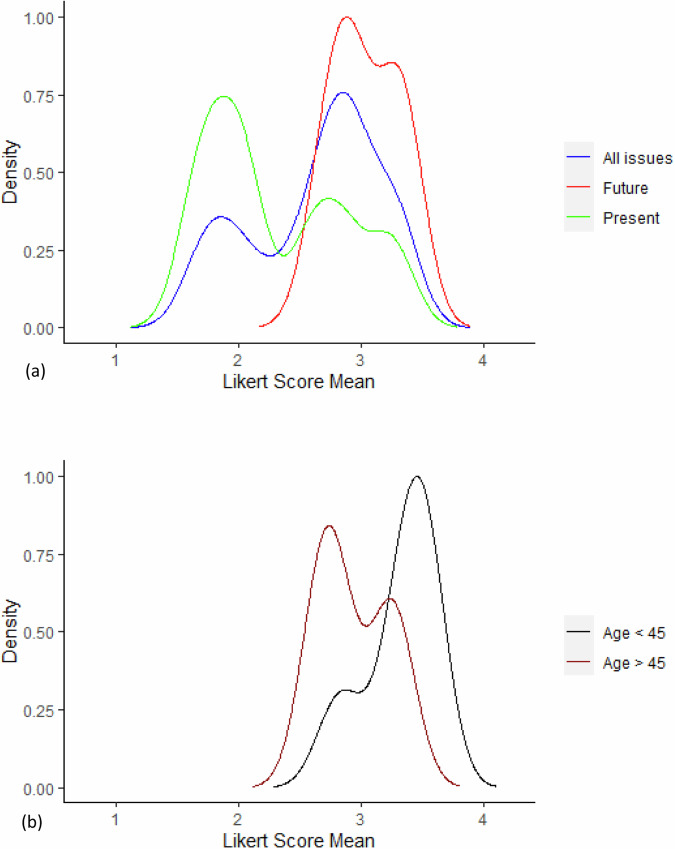
Density plots of Likert score means for Question 9 (How concerned are you about the following factors that can be involved in living with cardiomyopathy). **a** degree of concern among all respondents about all 20 issues (blue), seven ‘present’ (green), and five ‘future’ (red) issues; **b** degree of concern about five ‘future’ issues among respondents age 45 and under (black), and over age 45 (dark red).

Comparing mean Likert scores for present-only with future-only issues showed significantly greater concern for future-only issues (Fig. 3a; mean Likert score 2.31 for present and 3.04 for future issues, *p* = 0.0303). Concern about issues with a future-only dimension was significantly greater among respondents aged 45 and under, than for respondents aged over 45 (mean Likert concern 3.32 for age <45 and 2.94 for age >45, *p* = 0.047; Fig. 3b and Table S1). For issues in the category “both present and future”, concern was also significantly greater among respondents aged 45 and under (*p* = 0.021), and concern across all issues was significantly greater in respondents age <45 (*p* = 0.023). The difference in distribution of concern about future issues between females and males was not significant (*p* = 0.25).

In total, 150 respondents provided free text responses about issues that can be involved in living with cardiomyopathy. Responses related to three main themes: psychosocial concerns; symptom-related concerns; medical care. A fourth theme relates to views about genetic therapy for cardiomyopathy. Further examples of free-text comments, organized by theme, are shown in Table S2.

### Psychosocial concerns: family

Many respondents cited individual family members affected, with some outlining the range of manifestations experienced including sudden or premature death, heart transplant, ICD, heart failure, atrial fibrillation, stroke. Adverse events in one or more relative resulted in, or exacerbated, long-term anxiety about other relatives: *I am G* + *P* + [assumed “genotype positive, phenotype positive”]*. 2 of my 3 children inherited my G* + *. The HCM-related symptoms are hell to live with and the death of a child simply breaks families. My husband & I are broken people*. (F age 56–65, USA. HCM)

For some, the impact of a close relative’s sudden death influenced thoughts about future life plans and potential trial participation: *My mum passed away when I was 14 as a result of a cardiac arrest from HCM. I can’t think about my future (having kids, having a stressful job, keeping fit) without thinking I will die young. If this may be available in the future to help my children who may inherit it, it would be a great opportunity to think of trying this myself*. (F age 16–25, UK. HCM)

Some respondents described concern as “huge” or “extreme”. Many elaborated on concerns that related primarily to their children and/or grandchildren; concern was irrespective of descendants’ age, clinical or genetic test status (if children were not yet tested). A small number commented that concerns were influencing decisions about having children or had decided not to have children to avoid transmission. Some perceived that anxiety affected the whole family: *My … son had a cardiac arrest at school …, thankfully his teachers saved his life. [He] was fitted with a ICD and has since had 4 more cardiac arrests. I have passed the genetic change onto my daughter … the future is scary for all of us and the everyday worries is heart wrenching*. (F age 26–35, UK. HCM).

### Psychosocial concerns: personal uncertainty

Respondents wrote about worry, anxiety or fear about sudden death, syncope, disease progression, need for medical intervention, repeated hospitalizations and the impacts on many aspects of life, including safe levels of exercise, impacts on lifestyle/employment, and life expectancy. Uncertainty about disease manifestation was a concern for variant carriers.

### Symptom-related concerns

Respondents wrote about physical impacts including symptoms of heart failure, syncope, and medical interventions including ICD, myectomy, ablation, medication and (need for) cardiac transplant: *All of these concerns* [i.e. listed in Q9] *were present before my transplant, my new concern is how long I will live for*. (F age 16–25, UK. RCM).

Several mentioned social perceptions, such as “Others not understanding how symptoms can make you feel day-to-day especially as you may not look unwell visually” (F age 46–55, UK. DCM) and concerns about disability benefits.

Respondents often linked disease complications with psychological consequences and family responsibilities: *I have arvd…..just been turned down for transplant…. I am desperate for help as I need to be around longer for my young family* (M age 46–55, UK. ACM).

### Medical care and genetic testing

Although some mentioned that genetic testing had been instrumental in identifying asymptomatic relatives, a small number mentioned that genetic testing had not been offered or was contingent on additional relatives developing a phenotype. For respondents in whose family no genetic cause had been found, the need for continued clinical screening was a concern. Comments about medical care included perceived limited knowledge or understanding among healthcare professionals, differences of opinion among specialists, imperfect communication, difficulties with pediatric to adult transition, and strained healthcare resources. Several mentioned difficulties accessing screening for relatives: *My son died from HCM at the age of 36. We have not been offered any medical follow up or monitoring for [his descendants]. I am very anxious about this* (F age 66–75, UK. Relative with HCM).

### Attitudes to gene therapy

Ninety-three percent had watched the explanatory video, and 80.1% thought they had a better understanding of gene therapy after watching the video. In response to the question “Do you think gene therapy should be developed for use in cardiomyopathy”, 93.6% answered yes; 6.4% said they did not know.

To understand respondents’ attitudes to application of gene therapy according to clinical presentation and age, the survey presented six scenarios (Table 1), varying by age and clinical factors and asked respondents to choose whether, in that scenario, they would be interested in taking part in a gene therapy trial, if available. Individuals represented by Scenarios C and D may be asymptomatic, but cardiac testing may have shown risk of potentially life-threatening arrythmia warranting exercise restriction and implantation of an ICD. An ICD connects to the heart using thin wires and can detect and respond to arrhythmias with electrical signals to restore normal cardiac rhythm.

**Table 1:** Scenarios presented in survey.

Scenario	Description
F	Alex is a child age 5 who has no symptoms and is very well. However, Alex’s brother died recently from cardiomyopathy aged 7. A genetic variant, thought to be the cause of cardiomyopathy, was found in his brother. Alex carries the same genetic variant
E	Jasmine is a child age 5 who has cardiomyopathy. Her symptoms affect her everyday life
D	Jo is age 50. She has cardiomyopathy. Her symptoms do not affect her everyday life, but her doctor has advised her to limit her exercise and consider an implantable cardioverter defibrillator (ICD) because she is at risk of dangerous arrhythmia
C	John is age 20. He has cardiomyopathy. His symptoms do not affect his everyday life, but his doctor has advised him to limit his exercise and consider an implantable cardioverter defibrillator (ICD) because he is at risk of dangerous arrhythmia (abnormal heart rhythm)
B	Faizan is age 50 and has cardiomyopathy. His symptoms affect his everyday life. His doctor has told him that his condition has progressed over recent years
A	Mary is age 20. She has cardiomyopathy and her symptoms affect her everyday life. Her doctor has told her that her condition has progressed (worsened) over recent years

**Fig. 4:**
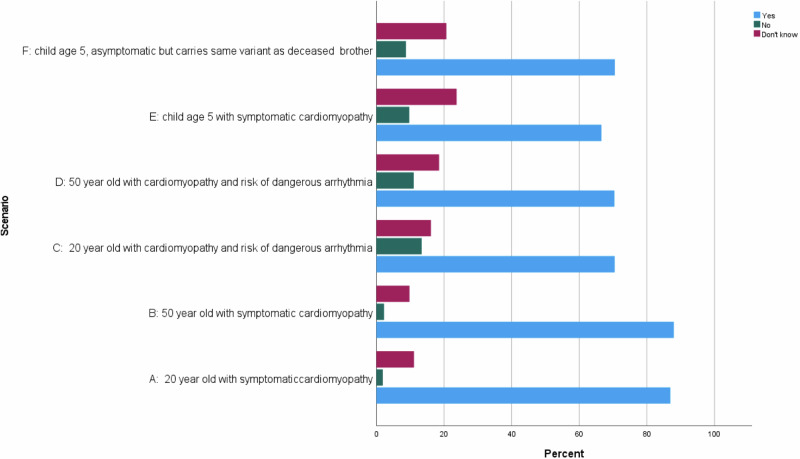
Willingness to consider participation in a gene therapy trial for cardiomyopathy. Proportion of respondents who would consider participation in a gene therapy trial for cardiomyopathy in the six scenarios shown in Table 1.

For all scenarios, more than 60% of respondents responded that they would consider trial participation (Fig. 4); significantly more respondents indicated that they would consider participation in scenarios indicating intrusive symptoms and disease progression (A and B) than scenarios with sudden death risk but asymptomatic (C and D; *p* value < 0.05) or a young child (E and F; *p* value < 0.05). The difference between scenarios C and D, and E and F was not significant.

**Fig. 5:**
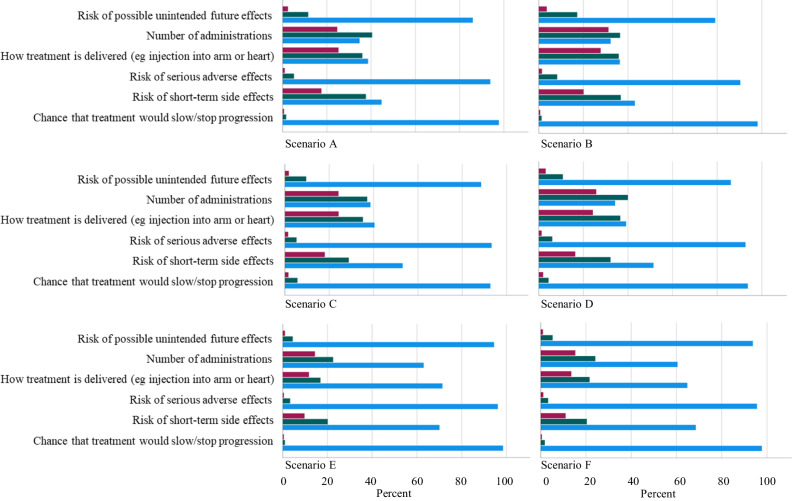
Importance of gene therapy attributes in each scenario. Graphs show the proportion of respondents who answered ‘important’ (blue); ‘neither important nor unimportant’ (green); ‘not important’ (purple) to the attributes shown, in the scenarios shown in Table 1.

To understand respondents’ attitudes to potential treatment risks, mode and route of treatment administration in relation to clinical presentation and age, respondents were asked how important a range of factors were in deciding whether to take part in a trial: *the chance that the treatment would stop or slow cardiomyopathy progression*; *the risk of short term side effects; the risk of serious adverse effects*; *how the treatment is delivered (for example as an injection in the arm, or directly into the heart*); *the number of times the treatment needs to be given (for example once only, or repeated twice a year*); *the risk of possible unintended future effects*. For all scenarios, the majority of respondents said *the chance that the treatment would stop or slow cardiomyopathy progression, risk of unintended future effects*, and *risk of serious adverse effects* was “important”. In all adult scenarios (A, B, C, D), fewer respondents considered that ‘the number of adminstrations’, and ‘how the treatment is delivered’ were important (Fig. 5, blue bars).

Among free-text comments in the theme *views of gene therapy for cardiomyopathy* (Table S2), the majority were positive, with some stating they would like to be involved. Several referred to their families’ long experience of cardiomyopathy: *As a family with 3 generations of people affected with arvc and gene positive I hope to see gene therapy progress so the 4th generation have a chance of a life without arvc* F age 46–55, UK. ARVC.

Some expressed nuanced views about risks and benefits, prompted by consideration of survey scenarios: without understanding the penetrance of the gene involved, I can’t put myself in the position of the parent whether or not to allow my 5 year old to be involved. I’d consider the trial and would want to find out but I would weigh out the risks for my child. Similarly, what benefit does a 50 year old get if cardiac remodeling has already happened and this is unlikely to be reversed unless activation of satellite cells occurs? I would be much more likely to participate as a 50 year old than as one of my children though (M age 36–45, UK. HCM).

Deciding to take part in gene therapy trials was seen by some as less complex for middle-aged to older adults. Some did not favor child participation, for ethical reasons, availability of alternatives, and funding prioritization: *Question 18 is excessive and unnecessarily expensive. The child has no symptoms and can be routinely checked & scanned. This type of therapy should only be used for those with symptoms. Funding is not infinite. [Public health funding] can only cover so much and having everyone who has a faulty gene expecting treatment with or without symptoms is a slippery slope* (F age 36–45, UK. HCM).

Some weighed genetic therapies against the need for, and limitations of, conventional treatment, and some commented that they would need more information about the risks, and/or preliminary trial results before making a decision.

## Discussion

This is the first report of attitudes toward genetic therapies for cardiomyopathy among the patient community. Our survey of over 600 respondents, the vast majority with personal and/or family history of cardiomyopathy, finds overwhelming support for the development of genetic therapy tools and strategies. Survey findings support studies showing that cardiomyopathy disease burden is considerable [7–9, 11, 14, 20, 21], with many respondents reporting significant psychosocial concerns irrespective of symptoms. Free text responses indicate the important contribution of family experience to perceptions of cardiomyopathy, its future progression, and worry about other relatives. Concern about issues with a future dimension is significantly greater than concern about issues affecting people at the present time, and younger respondents had significantly more concern about issues with a future dimension than older respondents.

The survey finds that psychosocial concerns are an important component of disease burden of cardiomyopathy; anxiety about the future with cardiomyopathy is high, for individuals and for their existing and potential children. Family members’ experiences of cardiomyopathy influence individuals’ perceptions of how cardiomyopathy might progress. From a healthcare perspective, our data support provision of patient access to psychosocial support including but not limited to genetic counseling, in line with recent recommendations [24]. Pre-requisites for equitable access to future genetic therapies—and realizing the hopes of the patient community—are consistent and equitable access to genetic testing, and educating specialist health professionals about emerging therapies.

From the perspective of genetic therapy development, understanding the potential benefits for cost effectiveness analyses and regulatory approval will require patient-reported outcomes measures (PROMs) that fully capture cardiomyopathy disease burden. Genetic therapies may become applicable to individuals before they have developed advanced cardiomyopathy, and PROMs of disease burden that prioritize symptoms and physical limitations, such as the Kansas City Cardiomyopathy Questionnaire [33] are inadequate. Comprehensive understanding of disease burden should include the pre-symptomatic phase and incorporate the familial nature of cardiomyopathy.

Clinical manifestations of cardiomyopathy and age at onset are highly variable within and between families, and application of current management tools depends largely on disease expressivity (for example treatment of heart failure symptoms, or SCD risk). Survey scenario responses show that members of the community take this variability, and its implications, into account when considering acceptability of genetic therapies. Free-text responses also suggest that family members’ disease experience might influence decisions about trial participation, and it will be important to consider the extent to which psychosocial factors should play a role in patient selection and acceptance. This will be especially important if trials recruit children. Respondents indicate considered and nuanced attitudes toward the risk/benefit ratio, mode and frequency of therapy administration, and how risk/benefit considerations might vary according to age and clinical situation.

In their recent framework for human genome editing governance, the World Health Organization recommend developing governance structures that ensure accountability, transparency, responsiveness, equity and inclusiveness, and broad-based participation [34]. Patient and public support is critical for the viability of any new technology, and benefits must be balanced against a range of possible personal and societal harms. Implementation of genetic therapies will require trust, fostered by effective education and transparency [35]. Allyse et al. [36] use the term “translational justice” to describe the active and integrative involvement of end-user groups in human genome editing policy development, arguing that ethical translation should equitably address the values and needs of affected stakeholders including, but not limited to, patient communities. This preliminary study forms the basis for our future work to promote ethical translation of genetic therapies for cardiomyopathy.

### Limitations and further research

The survey was designed to understand preliminary attitudes to gene therapy and was accompanied by an information video introducing key concepts. It was not designed to allow generation of statistical correlations between clinical situations or disease family history, with attitudes. We did not collect data about respondents’ current symptoms, since we did not aim to correlate symptoms with responses to other questions. Our results suggest that factors associated with clinical situation and family history will influence willingness to participate in genetic therapy trials, and further research is needed to understand correlations.

## Conclusions

Our survey, designed to understand preliminary attitudes to genetic therapies for cardiomyopathy, found that the vast majority of the patient community support development. Concern about cardiomyopathy-related issues with a future dimension, such as disease progression, was significantly greater than concern about current issues, and concern was often informed by family experience. A comprehensive understanding of the burden of cardiomyopathy will inform development and future implementation of genetic therapies.

### Supplementary information

Survey

Analysis of How concerned are you about the following factors that can be involved in living with cardiomyopathy? (Question 9)

Illustrative free-text comments (reproduced verbatim) provided by survey respondents, by theme.

## Data Availability

Survey response data are available on reasonable request to the corresponding author.

## References

[1] Watkins H, Ashrafian H, Redwood C. Inherited cardiomyopathies. N Engl J Med. 2011;364:1643–56. 21524215 10.1056/NEJMra0902923

[2] Semsarian C, Ingles J, Maron MS, Maron BJ. New perspectives on the prevalence of hypertrophic cardiomyopathy. J Am Coll Cardiol. 2015;65:1249–54. 25814232 10.1016/j.jacc.2015.01.019

[3] Hershberger RE, Hedges DJ, Morales A. Dilated cardiomyopathy: the complexity of a diverse genetic architecture. Nat Rev Cardiol. 2013;10:531–47. 23900355 10.1038/nrcardio.2013.105

[4] Ho CY, Day SM, Ashley EA, Michels M, Pereira AC, Jacoby D, et al. Genotype and lifetime burden of disease in hypertrophic cardiomyopathy. Circulation. 2018;138:1387–98. 30297972 10.1161/CIRCULATIONAHA.117.033200 PMC6170149

[5] Yotti R, Seidman CE, Seidman JG. Advances in the genetic basis and pathogenesis of sarcomere cardiomyopathies. Annu Rev Genomics Hum Genet. 2019;20:129–53. 30978303 10.1146/annurev-genom-083118-015306

[6] Ware JS, Cook SA. Role of titin in cardiomyopathy: from DNA variants to patient stratification. Nat Rev Cardiol. 2018;15:241–52. 29238064 10.1038/nrcardio.2017.190

[7] Zaiser E, Sehnert AJ, Duenas A, Saberi S, Brookes E, Reaney M. Patient experiences with hypertrophic cardiomyopathy: a conceptual model of symptoms and impacts on quality of life. J Patient Rep. Outcomes. 2020;4:102. 33259041 10.1186/s41687-020-00269-8 PMC7708573

[8] Cox S, O’Donoghue AC, McKenna WJ, Steptoe A. Health related quality of life and psychological wellbeing in patients with hypertrophic cardiomyopathy. Heart Br Card Soc. 1997;78:182–7. 10.1136/hrt.78.2.182 PMC484901 9326995

[9] Hidayatallah N, Silverstein LB, Stolerman M, McDonald T, Walsh CA, Paljevic E, et al. Psychological stress associated with cardiogenetic conditions. Pers Med. 2014;11:631–40. 10.2217/pme.14.50 PMC4242419 25431604

[10] Rhodes AC, Murray B, Tichnell C, James CA, Calkins H, Sears SF. Quality of life metrics in arrhythmogenic right ventricular cardiomyopathy patients: the impact of age, shock and sex. Int J Cardiol. 2017;248:216–20. 28823501 10.1016/j.ijcard.2017.08.026

[11] Ingles J, Spinks C, Yeates L, McGeechan K, Kasparian N, Semsarian C. Posttraumatic stress and prolonged grief after the sudden cardiac death of a young relative. JAMA Intern Med. 2016;176:402–5. 26809585 10.1001/jamainternmed.2015.7808

[12] Yeates L, Hunt L, Saleh M, Semsarian C, Ingles J. Poor psychological wellbeing particularly in mothers following sudden cardiac death in the young. Eur J Cardiovasc Nurs. 2013;12:484–91. 23568895 10.1177/1474515113485510

[13] van der Werf C, Onderwater AT, van Langen IM, Smets EMA. Experiences, considerations and emotions relating to cardiogenetic evaluation in relatives of young sudden cardiac death victims. Eur J Hum Genet. 2014;22:192–6. 23736216 10.1038/ejhg.2013.126 PMC3895645

[14] Bates K, Sweeting J, Yeates L, McDonald K, Semsarian C, Ingles J. Psychological adaptation to molecular autopsy findings following sudden cardiac death in the young. Genet Med J Am Coll Med Genet. 2019;21:1452–6. 10.1038/s41436-018-0338-4 30327538

[15] Bezzina CR, Lahrouchi N, Priori SG. Genetics of sudden cardiac death. Circ Res. 2015;116:1919–36. 26044248 10.1161/CIRCRESAHA.116.304030

[16] Walsh R, Thomson KL, Ware JS, Funke BH, Woodley J, McGuire KJ, et al. Reassessment of Mendelian gene pathogenicity using 7,855 cardiomyopathy cases and 60,706 reference samples. Genet Med J Am Coll Med Genet. 2017;19:192–203. 10.1038/gim.2016.90 PMC5116235 27532257

[17] Harper AR, Goel A, Grace C, Thomson KL, Petersen SE, Xu X, et al. Common genetic variants and modifiable risk factors underpin hypertrophic cardiomyopathy susceptibility and expressivity. Nat Genet. 2021;53:135–42. 33495597 10.1038/s41588-020-00764-0 PMC8240954

[18] Tadros R, Francis C, Xu X, Vermeer AMC, Harper AR, Huurman R, et al. Shared genetic pathways contribute to risk of hypertrophic and dilated cardiomyopathies with opposite directions of effect. Nat Genet. 2021;53:128–34. 33495596 10.1038/s41588-020-00762-2 PMC7611259

[19] Wilde AAM, Semsarian C, Márquez MF, Sepehri Shamloo A, Ackerman MJ, Ashley EA, et al. European Heart Rhythm Association (EHRA)/Heart Rhythm Society (HRS)/Asia Pacific Heart Rhythm Society (APHRS)/Latin American Heart Rhythm Society (LAHRS) Expert Consensus Statement on the state of genetic testing for cardiac diseases. J Arrhythmia. 2022;38:491–553. 10.1002/joa3.12717 PMC9347209 35936045

[20] Ormondroyd E, Oates S, Parker M, Blair E, Watkins H. Pre-symptomatic genetic testing for inherited cardiac conditions: a qualitative exploration of psychosocial and ethical implications. Eur J Hum Genet. 2014;22:88–93. 23632793 10.1038/ejhg.2013.81 PMC3865409

[21] Bonner C, Spinks C, Semsarian C, Barratt A, Ingles J, McCaffery K. Psychosocial impact of a positive gene result for asymptomatic relatives at risk of hypertrophic cardiomyopathy. J Genet Couns. 2018;27:1040–8. 29468337 10.1007/s10897-018-0218-8

[22] Bordet C, Brice S, Maupain C, Gandjbakhch E, Isidor B, Palmyre A, et al. Psychosocial impact of predictive genetic testing in hereditary heart diseases: the PREDICT study. J Clin Med. 2020;9:1365. 32384747 10.3390/jcm9051365 PMC7290753

[23] Authors/Task Force Members, Priori SG, Blomström-Lundqvist C, Mazzanti A, Blom N, Borggrefe M, et al. 2015 ESC guidelines for the management of patients with ventricular arrhythmias and the prevention of sudden cardiac death: the Task Force for the Management of Patients with Ventricular Arrhythmias and the Prevention of Sudden Cardiac Death of the European Society of Cardiology (ESC)Endorsed by: Association for European Paediatric and Congenital Cardiology (AEPC). EP Eur. 2015;17:1601–87. 10.1093/europace/euv319 26318695

[24] Arbelo E, Protonotarios A, Gimeno JR, Arbustini E, Barriales-Villa R, Basso C, et al. 2023 ESC guidelines for the management of cardiomyopathies. Eur Heart J. 2023;44:3503–626. 37622657 10.1093/eurheartj/ehad194

[25] Anguela XM, High KA. Entering the modern era of gene therapy. Annu Rev Med. 2019;70:273–88. 30477394 10.1146/annurev-med-012017-043332

[26] Reichart D, Newby GA, Wakimoto H, Lun M, Gorham JM, Curran JJ, et al. Efficient in vivo genome editing prevents hypertrophic cardiomyopathy in mice. Nat Med. 2023;29:412–21. 36797483 10.1038/s41591-022-02190-7 PMC9941048

[27] Chai AC, Cui M, Chemello F, Li H, Chen K, Tan W, et al. Base editing correction of hypertrophic cardiomyopathy in human cardiomyocytes and humanized mice. Nat Med. 2023;29:401–11. 36797478 10.1038/s41591-022-02176-5 PMC10053064

[28] Argiro A, Bui Q, Hong KN, Ammirati E, Olivotto I, Adler E. Applications of gene therapy in cardiomyopathies. JACC Heart Fail. 2024;12:248–60. 10.1016/j.jchf.2023.09.015 37966402

[29] Helms AS, Thompson AD, Day SM. Translation of new and emerging therapies for genetic cardiomyopathies. JACC Basic Transl Sci. 2022;7:70. 35128211 10.1016/j.jacbts.2021.07.012 PMC8807730

[30] Cornel MC, Howard HC, Lim D, Bonham VL, Wartiovaara K. Moving towards a cure in genetics: what is needed to bring somatic gene therapy to the clinic? Eur J Hum Genet. 2019;27:484–7. 30568241 10.1038/s41431-018-0309-x PMC6460577

[31] Avkiran M. CureHeart wins Big Beat Challenge, a £30 million research award from the British Heart Foundation. Eur Heart J. 2022;43:4450–2. 36151852 10.1093/eurheartj/ehac510 PMC9637421

[32] Braun V, Clarke V. Using thematic analysis in psychology. Qual Res Psychol. 2006;3:77–101. 10.1191/1478088706qp063oa

[33] Green CP, Porter CB, Bresnahan DR, Spertus JA. Development and evaluation of the Kansas City Cardiomyopathy Questionnaire: a new health status measure for heart failure. J Am Coll Cardiol. 2000;35:1245–55. 10758967 10.1016/S0735-1097(00)00531-3

[34] World Health Organisation. Human genome editing: a framework for governance. 2023. https://www.who.int/publications-detail-redirect/9789240030060.

[35] Delhove J, Osenk I, Prichard I, Donnelley M. Public acceptability of gene therapy and gene editing for human use: a systematic review. Hum Gene Ther. 2020;31:20–46. 31802714 10.1089/hum.2019.197

[36] Allyse MA, Meagher KM, Michie M, Isasi R, Ormond KE, Bonhomme N, et al. Translational justice in human gene editing: bringing end user engagement and policy together. Am J Bioeth. 2023;23:55–8. 37339310 10.1080/15265161.2023.2207513 PMC10441003

