# Supplementary material for: Genetic therapies for cardiomyopathy: survey of attitudes of the patient community for the CureHeart project
Source: Eur J Hum Genet. 2024 Jul 7;32(9):1045–52. doi: 10.1038/s41431-024-01660-5 (PMC11368914; doi:10.1038/s41431-024-01660-5)
Supplement: Supplementary file 1 — Survey [file 41431_2024_1660_MOESM1_ESM.pdf]

CureHeart survey

Page 1: Page 1

We invite you to participate in a survey about gene therapy for cardiomyopathy, conducted by researchers at the University of Oxford. The information generated will be used to inform a bid for funding from the British Heart Foundation.

The study we propose aims to develop treatments that would correct the genetic alterations in the heart that cause cardiomyopathy, and so provide the first cures for these conditions.

Taking this survey is completely voluntary. It should take about 10 minutes to complete. We will not ask your name or any identifying information.

By completing this survey, you are consenting to the use of the information you provide, on the understanding that it will be aggregated for purposes of analysis, publications, and presentations. Your responses will not be identified or identifiable.

We may use any free text as quotes in publicity materials, but it will not be possible to identify the respondent, and we will take care not to include any quotes which indicate personal context.

Please watch the CureHeart video before you start the survey. It contains information about the treatment we want to develop.

1. I have watched the CureHeart video

- ○ Yes
- ○ No

Page 2: Questions

2. What is your experience of cardiomyopathy? (you may choose more than one)

- ☐ I have cardiomyopathy
- ☐ Someone in my family has cardiomyopathy
- ☐ Someone I know well (who is not in my family) has cardiomyopathy
- ☐ I don’t have cardiomyopathy, and I don’t know anyone who has cardiomyopathy

3. If you are affected with cardiomyopathy or know someone who is, which cardiomyopathy do you, or they, have? (please choose one)

- ○ Hypertrophic cardiomyopathy (HCM)
- ○ Dilated cardiomyopathy (DCM)
- ○ Arrhythmogenic right ventricular cardiomyopathy (ARVC or ACM)
- ○ Restrictive cardiomyopathy (RCM)
- ○ Left ventricular non compaction cardiomyopathy (LVNC)
- ○ Other cardiomyopathy
- ○ Don’t know
- ○ Not applicable - I don't have, or know anyone who has cardiomyopathy

4. Have you, or someone you know who has cardiomyoapthy, had genetic testing for cardiomyopathy? (please choose one)

- ○ Yes, genetic testing has been done
- ○ No, genetic testing was offered but declined
- ○ No, genetic testing has not been offered
- ○ Don’t know
- ○ Not applicable – I don’t have or know anyone with cardiomyopathy

5. What is your age?

- 15 or younger
- 16-25
- 26-35
- 36-45
- 46-55
- 56-65
- 66-75
- 76 or over

6. What gender do you identify as?

- Female
- Male
- Non-binary

7. Where do you live?

- UK
- USA
- Other

7.a. Please state country or region

8. Do you identify as an ethnic minority?

- ○ Yes
- ○ No
- ○ Don't know

9. How concerned are you about the following factors that can be involved in living with cardiomyopathy?

Please don't select more than 1 answer(s) per row.

	Not concerned	slightly concerned	quite concerned	very concerned
How the cardiomyopathy might progress in future	☐	☐	☐	☐
Uncertainty about whether I, or someone I know, has cardiomyopathy	☐	☐	☐	☐
Whether my children, or the children of someone I know, might develop cardiomyopathy in future	☐	☐	☐	☐
Having symptoms that interfere with quality of life (for example, blackouts or breathlessness)	☐	☐	☐	☐
Side effects of medication	☐	☐	☐	☐
The need to take medication long term (for many years)	☐	☐	☐	☐
Lifestyle advice (for example, needing to limit exercise or stop doing certain activities)	☐	☐	☐	☐

Whether an implantable cardioverter defibrillator (ICD) is, or might be needed	☐	☐	☐	☐
Problems with existing ICD	☐	☐	☐	☐
How to get genetic testing	☐	☐	☐	☐
Whether genetic testing is a good idea	☐	☐	☐	☐
What genetic test results mean	☐	☐	☐	☐
Whether to become pregnant (if female) or whether my relative or partner should become pregnant	☐	☐	☐	☐
Passing on cardiomyopathy to future children	☐	☐	☐	☐
Effects on current job or future career choice	☐	☐	☐	☐
Effects on other areas of life (for example spare time activities)	☐	☐	☐	☐
Effects on mental health	☐	☐	☐	☐
The risk of dying suddenly	☐	☐	☐	☐
The possibility of needing a heart transplant in future	☐	☐	☐	☐
Getting insurance	☐	☐	☐	☐

9.a. Do you want to add any other concerns, or tell us more?

10. **Had you heard about gene therapy before watching the video?**

- Yes
- No
- Don't know

11. **Do you think you have a better understanding of gene therapy after watching the video?**

- Yes
- No
- Don't know
- Not applicable - I haven't watched the video

12. **Do you think gene therapy should be developed for use in cardiomyopathy?**

- Yes
- No
- Don't know

Please read and think about the following 6 scenarios, and answer the questions which follow them. In each scenario, the person who has cardiomyopathy has a genetic variant considered to be the cause of cardiomyopathy.

13. **Scenario A**: *Mary is age 20. She has cardiomyopathy and her symptoms affect her everyday life. Her doctor has told her that her condition has progressed (worsened) over recent years.* **If you were Mary, and if a gene therapy trial were available do you think you would be interested in taking part?**

- Yes
- No
- Don't know

13.a. **If you were Mary, and if a gene therapy trial were available how important would the following factors be in your decision to take part?**

Please don't select more than 1 answer(s) per row.

	important	neither important not unimportant	not important
The chance that the treatment would stop or slow cardiomyopathy progression	☐	☐	☐
The risk of short term side effects	☐	☐	☐
The risk of serious adverse effects	☐	☐	☐
How the treatment is delivered (for example as an injection in the arm, or directly into the heart)	☐	☐	☐
The number of times the treatment needs to be given (for example once only, or repeated twice a year)	☐	☐	☐
The risk of possible unintended future effects	☐	☐	☐

14. **Scenario B**: *Faizan is age 50 and has cardiomyopathy. His symptoms affect his everyday life. His doctor has told him that his condition has progressed over recent years.* **If you were Faizan, and if a gene therapy trial were available do you think you would be interested in taking part?**

- Yes
- No
- Don't know

14.a. If you were Faizan, and if a gene therapy trial were available how important would the following factors be in your decision to take part?

Please don't select more than 1 answer(s) per row.

	important	neither important not unimportant	not important
The chance that the treatment would stop or slow cardiomyopathy progression	☐	☐	☐
The risk of short term side effects	☐	☐	☐
The risk of serious adverse effects	☐	☐	☐
How the treatment is delivered (for example as an injection in the arm, or directly into the heart)	☐	☐	☐
The number of times the treatment needs to be given (for example once only, or repeated twice a year)	☐	☐	☐
The risk of possible unintended future effects	☐	☐	☐

15. **Scenario C***: John is age 20. He has cardiomyopathy. His symptoms do not affect his everyday life, but his doctor has advised him to limit his exercise and consider an implantable cardioverter defibrillator (ICD) because he is at risk of dangerous arrhythmia (abnormal heart rhythm).* **If you were John, and if a gene therapy trial were available do you think you would be interested in taking part?**

- ○ Yes
- ○ No
- ○ Don't know

15.a. If you were John, and if a gene therapy trial were available how important would the following factors be in your decision to take part?

Please don't select more than 1 answer(s) per row.

	important	neither important not unimportant	not important
The chance that the treatment would stop or slow cardiomyopathy progression	☐	☐	☐
The risk of short term side effects	☐	☐	☐
The risk of serious adverse effects	☐	☐	☐
How the treatment is delivered (for example as an injection in the arm, or directly into the heart)	☐	☐	☐
The number of times the treatment needs to be given (for example once only, or repeated twice a year)	☐	☐	☐
The risk of possible unintended future effects	☐	☐	☐

16. **Scenario D***: Jo is age 50. She has cardiomyopathy. Her symptoms do not affect her everyday life, but her doctor has advised her to limit her exercise and consider an implantable cardioverter defibrillator* (*ICD*) *because she is at risk of dangerous arrhythmia (abnormal heart rhythm).* **If you were Jo, and if a gene therapy trial were available do you think you would be interested in taking part?**

- ○ Yes
- ○ No
- ○ Don't know

16.a. **If you were Jo, and if a gene therapy trial were available how important would the following factors be in your decision to take part?**

Please don't select more than 1 answer(s) per row.

	important	neither important not unimportant	not important

The chance that the treatment would stop or slow cardiomyopathy progression	☐	☐	☐
The risk of short term side effects	☐	☐	☐
The risk of serious adverse effects	☐	☐	☐
How the treatment is delivered (for example as an injection in the arm, or directly into the heart)	☐	☐	☐
The number of times the treatment needs to be given (for example once only, or repeated twice a year)	☐	☐	☐
The risk of possible unintended future effects	☐	☐	☐

17. **Scenario E**: *Jasmine is a child age 5 who has cardiomyopathy. Her symptoms affect her everyday life.* **If you were Jasmine's parent, and if a gene therapy trial were available do you think you would be interested in Jasmine taking part?**

- ○ Yes
- ○ No
- ○ Don't know

17.a. **If you were Jasmine's parent, and if a gene therapy trial were available how important would the following factors be in your decision to take part?**

Please don't select more than 1 answer(s) per row.

	important	neither important not unimportant	not important
The chance that the treatment would stop or slow cardiomyopathy progression	☐	☐	☐
The risk of short term side effects	☐	☐	☐
The risk of serious adverse effects	☐	☐	☐

How the treatment is delivered (for example as an injection in the arm, or directly into the heart)	☐	☐	☐
The number of times the treatment needs to be given (for example once only, or repeated twice a year)	☐	☐	☐
The risk of possible unintended future effects	☐	☐	☐

18. **Scenario F:** *Alex is a child age 5 who has no symptoms and is very well. However, Alex's brother died recently from cardiomyopathy aged 7. A genetic variant, thought to be the cause of cardiomyopathy, was found in his brother. Alex carries the same genetic variant.* **If you were Alex's parent, and if a gene therapy trial were available do you think you would be interested in Alex taking part?**

- ◯ Yes
- ◯ No
- ◯ Don't know

18.a. **If you were Alex's parent, and if a gene therapy trial were available how important would the following factors be in your decision for Alex to take part?**

Please don't select more than 1 answer(s) per row.

	important	neither important not unimportant	not important
The chance that the treatment would stop or slow cardiomyopathy progression	☐	☐	☐
The risk of short term side effects	☐	☐	☐
The risk of serious adverse effects	☐	☐	☐
How the treatment is delivered (for example as an injection in the arm, or directly into the heart)	☐	☐	☐

The number of times the treatment needs to be given (for example once only, or repeated twice a year)	☐	☐	☐
The risk of possible unintended future effects	☐	☐	☐

19. Is there anything you wish to add?

Page 3: Final page

Thank you for taking time to answer this survey.

Your answers are important to us.

We hope to start trials within three to five years. At this early stage we're keen to learn the views of people with cardiomyopathy and their families, rather than looking for trial volunteers at present.
