# Supplementary material for: Genetic therapies for cardiomyopathy: survey of attitudes of the patient community for the CureHeart project
Source: Eur J Hum Genet. 2024 Jul 7;32(9):1045–52. doi: 10.1038/s41431-024-01660-5 (PMC11368914; doi:10.1038/s41431-024-01660-5)
Supplement: Supplementary file 2 — Analysis of How concerned are you about the following factors that can be involved in living with cardiomyopathy? (Question 9) [file 41431_2024_1660_MOESM2_ESM.docx]

**Table S1. Analysis of *How concerned are you about the following factors that can be involved in living with cardiomyopathy?*** (Question 9)
