# Supplementary material for: Genetic therapies for cardiomyopathy: survey of attitudes of the patient community for the CureHeart project
Source: Eur J Hum Genet. 2024 Jul 7;32(9):1045–52. doi: 10.1038/s41431-024-01660-5 (PMC11368914; doi:10.1038/s41431-024-01660-5)
Supplement: Supplementary file 3 — Illustrative free-text comments (reproduced verbatim) provided by survey respondents, by theme. [file 41431_2024_1660_MOESM3_ESM.docx]

| **Table S2. Illustrative free-text comments (reproduced verbatim) provided by survey respondents, by theme.** |
| --- |
| **Psychosocial concerns: family** |
| My partner has Hypertrophic cardiomyopathy and we decided to try for a baby. It worries me that my child could have this problem too but we don’t know any other way. We had a genetics test but have heard nothing else from them. (M age 16-25, UK. HCM) |
| I didn't get diagnosed till after having my children and I live my life with daily worries and what ifs (F age 36-45, UK. HCM) |
| My brothers and I have all said we don’t feel we can have children after my diagnosis. Our sister died waiting for heart transplant and now my diagnosis confirmed it was inherited. (F, age 26-35, UK. DCM) |
| My father has dilated cardiomyopathy & an ICD. Both myself & my sister have dilated cardiomyopathy. We all have the tnnt2 gene. My daughter died suddenly at 6 months old, her post mortem showed her heart was double the size it should of been. She was found to have dilated cardiomyopathy & the tnnt2 gene. (F age 26-35, UK. DCM) |
| I have tremendous guilt passing this gene to 3 of my children & 4 of my grandchildren (F age 66-75, USA. HCM) |
| I am awaiting a heart transplant and was only diagnosed in 2019. My concerns lie with my son possibly inheriting. (M, age 26-35, UK. HCM) |
| I was diagnosed at 38 my youngest son who is 17 just been diagnosed. I have a full family history of this disease, mum, uncle, cousin, grandad and his brother and daughter My eldest son keeps slipping through the net he’s 18 stuck between transition of paeds and adult services. (F age 36-45, UK. ACM) |
| I had HCM, for which I have received a heart transplant.. Unfortunately I have passed it onto my 14yr old daughter. (M, age 36-45, Ireland. HCM) |
| My brother died age 28 and now my son has discovered he has it aged 32. (F age 56-65, UK. HCM) |
| I do not have cardiomyopathy but my late husband and daughter had DCM. Also various other members of my husband's family, including his father and uncle and others who died before diagnosis. Both my husband and daughter had transplants. My daughter (aged 19 months) did not survive the operation, my husband had a further 12 years before his transplanted heart failed. He died at 43. My son was diagnosed as a baby with DCM and had a transplant at 18. He has recently (age 24) had surgery and is having chemo for intestinal lymphoma, caused by his immunosuppressant drugs.. His DCM was caused by a TNNT2 mutation. A cure for this disease would be incredible. (F age 56-65, UK. Relative with DCM) |
| **Psychosocial concerns: uncertainty** |
| Uncertainty, fear of death, my lifestyle being effected. (M age 16-25, UK. ACM) |
| As I have inherited the gene from my father, who has dilated cardiomyopathy I am uncertain about when this heart condition will prevail in me as I only have the gene. My grandfather and uncle have both passed away from it and I have checks every year at the royal Brompton hospital  (F age 16-25, UK. DCM) |
| Your life is just one big uncertainty. (F age 56-65, UK. DCM) |
| It has been a constant concern for the last 25 years as to when (or maybe if) all the problems begin. My Father suffered for years, then passed away aged just 57, I carry BAG 3 gene (F age 46-55, UK. DCM) |
| Concerns over the lack of answers on life expectancy. (M age 46-55, UK. LVNC) |
| Career restrictions. Not allowed to work front line services (police, fire, paramedic) in the uk (M age 26-35, UK. HCM) |
| **Symptom-related concerns** |
| Risk of atrial fibrillation, ablations, low flow state, hypotension from medications, LVAD for heart failure (F age 56-65, USA. HCM) |
| I am concerned about the risks of septal ablation and a myectomy if my condition were to deteriorate. (M age 66-75, UK. HCM) |
| Worried about day today tasks as getting harder to cope and what future holds (F age 56-65, UK. HCM) |
| “I worry about my daily decisions and their impacts” I worry I’ll have an “episode while driving” I worry if each day will be my last. (F age 56-65, USA. Other cardiomyopathy) |
| Concerns about syncope and its impact on job capacity is ever-present. (M age 36-45, USA. HCM) |
| **Medical care and genetic testing** |
| I’ve been under local hospital after being discharged from [hospital name] and wasn’t getting seen enough locally so now back under [hospital name] care. GP not helpful constantly tells you to go to A&E and won’t even take the time to learn or understand the disease. (F age 36-45, UK. ACM) |
| Poor lack of support in the nhs regarding the disease and doctors understand (M age 26-35, UK. HCM) |
| Getting treatment on the NHS after the covid-19 pandemic backlog. (F age 46-55, UK. DCM) |
| Getting the appropriate medical attention and full service response required, even if asymptomatic. (M age 26-35, UK. ACM) |
| Cheating the right medical advice as there seem to be a lot of shades of grey in the opinions of different specialists. Also making sure that data is properly shared between consultants. (M age 56-65, UK. LVNC) |
| Getting adequate and knowledgeable care when I need it. (F age 56-65, USA. HCM) |
| They can’t pinpoint the gene in my family yet so my biggest concern is the constant screening of me, my siblings and any future children I may have. (F age 26-35, UK. DCM) |
| **Views about genetic therapy for cardiomyopathy** |
| If I were a parent, I would do anything I could to ensure that my child survives. Do not underestimate the devastation caused by HCM related SCAs on the lives of families and communities. As an adult with HCM over the age of 50 & who lives with severe HCM symptoms, I would try gene therapy. If I were a parent of a HCM child, I would absolutely give gene therapy to my child. It is vastly different to be diagnosed with HCM as an adult who has already lived a full life of adventures than it is to be diagnosed as a child and told to restrict your activities & keep your heart rate down. HCM makes you afraid to live. We are meant to live full, rewarding lives & HCM prevents that. Before [adult child’s] death he had to give up sports and his dream of becoming a firefighter - both of which he trained for years and excelled at. HCM is a death sentence for dreams and aspirations. (F age 56-65, USA. HCM) |
| Treatment would give people hope. (F age 46-55, UK. ARVC) |
| In the final case, I would not want Alex to take part unless he started to show symptoms which showed that he was expressing the gene (F age 66-75, UK. HCM) |
| I’m currently living healthily but know I have the gene and know how sudden this can affect you. If this may be available in the future to help my children who may inherit it, it would be a great opportunity to think of trying this myself if the opportunity ever arises. I want to thank you for your interest in this work because I hope my kids never have to go through losing their mum like I did. This research gives me hope. (F age 16-25, UK. HCM) |
| My husband has arvc (42) and my son carries the gene (10 months old) we want to find out if we can take part in the trial. (F age 36-45, UK. Relative with ARVC) |
| I think an older person would be more inclined to take part in trials as they don't usually have so many people dependent on them if things went wrong. (F age 66-75, UK. DCM) |
| The uncertainty of living with DCM / ARVC affects every aspect of my life. This study is so exciting because it promises so much more than the other small, incremental improvements offered by current medication, ICDs, VADs and transplants. (F age 36-45, UK. DCM) |
| My son is 18 and has DCM. He is asymptomatic. I would not want him to participate in a gene therapy trial if there were risks involved. But I required a heart transplant at a relatively young age. If his condition deteriorated like mine did, then I would encourage him to participate in a gene therapy trial because in all likelihood he would also ultimately require a heart transplant. A heart transplant has given me the gift of life but is not a cure and not without risk. Gene therapy could be wonderful. (M age 56-65, UK. DCM) |
| There is always a risk benefit involved in any clinical trial. Therefore as much information as possible is needed. Are certain cardiomyopathy patients more likely to be benefit? e.g ARVC/D patient with certain defective genes or severity of myocyte damage. Or patients who are likely to parent children and want to protect future generations. (F age 36-45, UK. ARVC) |
| if there were adverse effects there is always the prospect of a transplant if one could be gotten soon enough before things became unmanageable. also would this prevent the gene from being passed on when the treatment paint bore children? (F age 76 or over, USA. HCM) |
